# Characteristics of Patients at First Visit to a Polio Clinic in Sweden

**DOI:** 10.1371/journal.pone.0150286

**Published:** 2016-03-16

**Authors:** Katarina Skough Vreede, Katharina S. Sunnerhagen

**Affiliations:** Department of Clinical Neuroscience, Institute of Neuroscience and Physiology The Sahlgrenska Academy, University of Gothenburg, Gothenburg, Sweden; IRCCS E. Medea, ITALY

## Abstract

**Aim:**

Describe polio patients visiting a polio clinic in Sweden, a country where vaccination was introduced in 1957.

**Design:**

A consecutive cohort study.

**Patients:**

Prior polio patients.

**Methods:**

All patients (n = 865) visiting the polio clinic at Sahlgrenska University Hospital, Gothenburg Sweden, between 1994 and 2012 were included in this study. Data at first visit regarding patient characteristics, polio classification, data of electromyography, origin, assistive devices and gait speed as well as muscle strength were collected for these patients. Twenty-three patients were excluded because no polio diagnosis could be established. A total of 842 patients with confirmed polio remained in the study.

**Results:**

More than twenty percent of the patients were from countries outside the Nordic region and considerably younger than those from the Nordic region. The majority of the emigrants were from Asia and Africa followed by Europe (outside the Nordic region). Of all patients included ninety-seven percent (n = 817) had polio in the lower extremity and almost 53% (n = 444) had polio in the upper extremity while 28% (n = 238) had polio in the trunk, according to clinical classification of polio. Compared with a sample of the normal population, the polio patients walked 61–71% slower, and were 53–77% weaker in muscle strength of the knee and foot as well as grip strength.

**Conclusion:**

The younger patients with polio emigrating from countries with different cultures may lead to a challenge for the multi professional teams working with post-polio rehabilitation and are of importance when planning for the care of polio patients the coming years.

## Introduction

A total of 12–20 million people worldwide have sequelae of poliomyelitis, according to Post-polio Health International [1]. Even today polio remains endemic in Afghanistan, Nigeria and Pakistan. Poliomyelitis is highly infectious and caused by one of three types of virus (type 1, 2 and 3), of which type 1 and 3 are still circulating in the endemic areas. However, only a small proportion of those infected get sequelae. The polio vaccine was introduced in the industrialized countries in the 1950s and 1960s. North- and South America have been polio free since 1994 and Europe since 2002. Polio can strike at any age but is most commonly seen in children, therefore polio are also called infantile paralysis. The virus enters the body through the mouth and multiplies in the intestine. Common symptoms of polio are fever, headache, fatigue, vomiting and stiffness in the neck. In some patients, the infections lead to affection of anterior horn cells resulting in irreversible flaccid paralysis usually in the legs [2]. After a period of clinical stability new or worsened symptoms may occur and are referred to as post-polio syndrome (PPS) [3–4]. Common symptoms of PPS are muscle weakness, fatigue, and muscle and/or joint pain [3, 5]. Little is known about the aftermaths of polio in large populations. Six hundred-eighteen polio patients in Boston were described in 1957 [6] and in 1998 a nation-wide survey of the medical and social situation of 1449 polio patients were performed in Norway [7]. In the Netherlands 260 polio survivors have been described regarding perceived health, disabilities and handicaps [8–9] and Windeback et al [10] have from 247 polio survivors selected 50 patients to describe in Olmstead County, Minnesota. In Sweden, 270 polio patients answered a study questionnaire in 1995 witch aimed to give an estimation of the patients’ current state of health [11].

The aim of the present study is to describe patients at first visit to a polio clinic in Sweden, a country where vaccination was introduced in 1957.

## Method

All patients (n = 865) visiting the polio clinic at Sahlgrenska University Hospital, Gothenburg Sweden, between 1994 and 2012 were included in this consecutive cohort study. Data regarding patient characteristics, polio classification and EMG data, origin, assistive devices and gait speed as well as muscle strength were collected for these patients. Twenty-three patients were excluded because no polio diagnosis could be established. A total of 842 patients with polio remained in the study.

### Measurements

#### Patient characteristics and classification of polio

Characteristic data, including clinical classification of polio, were collected by the patient’s physician at the polio clinic at Sahlgrenska University Hospital. The patients were also referred to analysis of electromyography (EMG) for verification and classification of polio.

#### Gait speed

The 30 meter Walk Test (WT), was used to evaluate gait speed [12–14]. First, the patients were instructed to walk at a speed they considered comfortable or normal (self-selected speed). Then, for maximum speed, they were asked to walk at a speed they considered to be as fast as possible, without running. The time taken to walk each distance was measured using a stopwatch, and the observer walked behind the patient without interfering.

#### Muscle strength of knee and foot

Measurements were performed on a Biodex® Multi-Joint System 3 PRO dynamometer. The equipment was calibrated before testing. The patient was seated comfortably with his or her back against a back rest. A seatbelt was strapped around the shoulders, waist and thigh to avoid unwanted movements. Before each measurement, the full range of motion was set and each patient’s lower extremity was weighed; the Biodex software corrected the data to account for the influence of the gravity effect torque on the data. The lower extremity was adjusted to the actuator with the axis of movement adjusted to center axis of the knee joint. Warm-up submaximal exercises were performed on a bicycle ergometer before the muscle test. The test order was randomized for both the paretic and non-paretic leg (drawing lot) to exclude learning effects. Maximal isokinetic muscle strength (in Newton meters, Nm) was measured at a velocity of 60°/sec during concentric muscle action of knee extensors and knee flexors for both lower extremities. This velocity has been found to be in accordance with the pedaling of the bicycle and the average velocity of the knee joint when walking [15]. Isometric knee extension and flexion strength was measured three times at 60° knee angle during 5 seconds in both legs.

Isometric endurance was measured as the time the patient was able to keep 40% of his/her isometric peak torque at 60° knee angle. The test was performed once. Isometric endurance was measured in seconds. A follow-up period of 5 minutes was used to evaluate the recovery process, with a maximum voluntary isometric contraction every minute (after 1, 2, 3, 4 and 5 minutes). The follow-up tests started less than 2 minutes after the endurance test. The recovery was measured as peak % of maximal result.

The peak isometric strength of foot dorsal flexors and plantar flexors at a 30° angle of knee flexion and a 0° ankle angle was also measured.

During the test, all patients were given visual feedback from the system’s monitor. They were also verbally encouraged by the examiner to make their maximal effort.

#### Hand grip

The handgrip force was assessed with Grippit^R^ [16], both the peak maximum grip force and the mean value of the ten-second sustained grip (both measured in Newton) for each hand. The person was seated on a chair without arm rests, with the lowest rib level with the edge of a table. The tested forearm was placed in the arm guide and the other arm rested on the table. The palm and fingers were completely clasped around the handle, and the force exerted against the transducer in the handle was recorded. The test has been shown to be reliable [17].

### Statistical analysis

The statistics program IBM SPSS Statistics version 22 (IBM Corp. Released 2013. IBM SPSS Statistics for Windows, Version 22.0. Armonk, NY: IBM Corp.) was used for the calculation of data. A *p*-value lesser than 0.05, was considered as statistically significant. Descriptive statistics (mean, SD, min/max) were used for patient characteristics, gait speed and results of muscle strength. Independent Sample test and Paired Samples T-test were used to analyse differences between different variables and groups. Data of isometric endurance in knee muscle where not normally distributed and therefore the non-parametric Mann-Whitney test was used. When analyzing the results of muscle strength, some patients had 0.0 (when the patient tried, but could not perform the test). These results were excluded in analysis. The values of the patients were normalized for age and sex based on control values of persons in the population ages 40–80 [18]. However, as some patients were younger than 40 years old and older than 79 years, the results are presented in age groups from -29 to 70- divided into groups of 20 years there between. When analyzing data, Turkey was included in Asia.

### Ethical approval

The study was approved by the Regional ethics committee in Western Sweden, http://www.epn.se/sv/goeteborg/om-naemnden/

Dnr 123–09. Data had been gathered for clinical use. The main aim of the registry is to facilitate quality improvement regarding polio care and to follow the process and all patients are informed that they can refuse data entering. Data was anonymized and de-identified prior to analysis

## Results

### Patient characteristics

Ninety-five percent (n = 801) of the 842 patients were diagnosed with PPS according to EFNS (European Federation of Neurological Society) [19]. Fifty-nine percent (n = 495) were female. For patient characteristics see Table 1.

**Table 1:** Patient characteristics of the patients included in the study.

	n	mean (SD)	min/max
Age (years)	842	59 (15)	14/93
TSO* (years)	842	52 (13)	8/85
Age at onset (years)	842	7 (8)	0/39
Height (m)	683	1.67 (0.09)	1.40/1.96
Weight (kg)	684	72 (14)	35/120
BMI**	662	26 (5)	14/44

*TSO = Time Since Onset of Polio

**BMI = Body Mass Index

### EMG and clinical classification of polio

Eighty-four percent (n = 708) of the patients completed examination by EMG in one or more extremities. One patient with no polio according to EMG was included in the study material due to clinical classified polio in right leg as well as poliovirus in spinal fluid. Seventy-eight percent (n = 656) of the patients included had polio verified by EMG in the lower extremities and 44% (n = 368) in the upper extremities when including all patients. Of those patients examined by EMG, in lower extremity/ies, ninety-seven percent (n = 656) had polio, and of those examined by EMG in upper extremity/ies 70% (n = 368) had polio.

**Fig 1:**
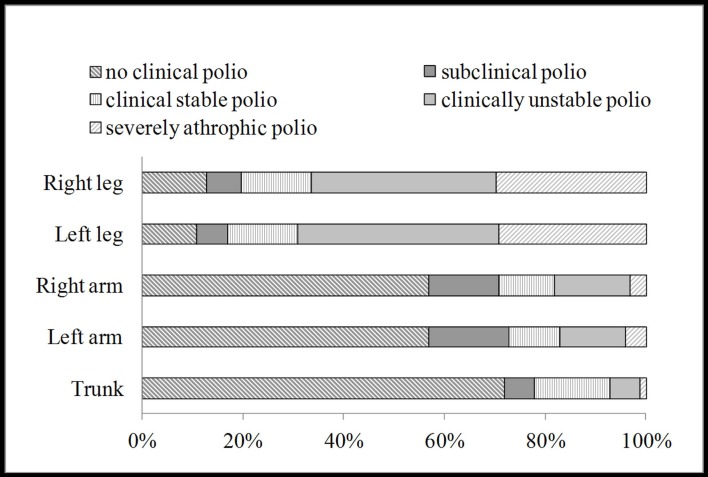
Clinical classification of polio (n = 842).

All patients included in the study had data from a clinical classification of polio. Ninety-seven percent (n = 817) had polio in the lower extremity and almost 53% (n = 444) had polio in the upper extremity while 28% (n = 238) had polio in the trunk. Data of body parts affected by polio shown for gender and Nordic region and outside Nordic region, respectively, see Table 2. For clinical classification of polio, see Fig 1.

**Table 2:** Polio according to clinical classification for women n = 495, men n = 347, total n = 842, Nordic region n = 662 and Outside Nordic region n = 180, respectively.

Body parts		women	men	total	Nordic region	Outside the Nordic region
Only right leg	n (%)	43 (9)	28 (8)	71 (8)	53 (8)	18 (10)
Only left leg	n (%)	59 (12)	27 (8)	86 (10)	69 (10)	17 (9)
Both legs	n (%)	375 (76)	285 (82)	660 (78)	515 (78)	145 (81)
No leg	n (%)	18 (4)	7 (2)	25 (3)	25 (4)	- (-)
Only right arm	n (%)	48 (10)	35 (10)	83 (10)	70 (11)	13 (7)
Only left arm	n (%)	51 (10)	30 (9)	81 (10)	64 (10)	17 (9)
Both arms	n (%)	143 (29)	137 (40)	280 (33)	223 (34)	57 (32)
No arm	n (%)	253 (51)	145 (42)	398 (47)	305 (46)	93 (52)

### Origin

Seventy-six percent (n = 639) of the patients were born in Sweden. When including all countries in the Nordic region the number increased marginally (to reach 79%, n = 662). Eighty-two percent (n = 690) of the patients were from Europe. Finland was the European country’s with the highest number of patients (n = 10) with sequelae of polio, immigrated to Sweden and visiting the post-polio outpatient clinic at Sahlgrenska University Hospital. From outside Europe, 66% of the patients were from Asia, 22% from Africa, 9% from South America and almost 3% were from North America. The countries outside Europe with the highest number of immigrating polio patients were Iraq (n = 34), Iran (n = 25), Somalia (n = 16), Turkey (n = 10), Lebanon (n = 9), Chile (n = 8) followed by Eritrea and India with seven immigrants each.

Women were dominating in the group of patients from the Nordic region (women n = 417, men n = 245). From Europe, outside The Nordic region, there were equal number of men and women (n = 14 of each). There were, however, more men than women from the group outside Europe (women n = 64, men n = 88).

Patients from the Nordic region were in average 8.5 years old at onset of polio, compared to patients from outside Nordic region who were in average 3.4 years old at onset of polio. The difference reached statistical significance (*p*<0.001). Patients coming from North America had the highest mean age of 9.8 years at onset of polio, followed by Europe 8.4 years, Africa 4.4 years, Asia 2.3 years and South America 2 years.

As seen in Table 3, patients from the Nordic region are in general much older than patients who have immigrated from outside Nordic region.

**Table 3:** Number of patients per age group divided into “Nordic region” (n = 622) and “Outside the Nordic region” (n = 180).

Age (years)		Nordic region	Outside the Nordic region
-29	n (%)	- (-)	43 (100)
30–49	n (%)	47 (33)	97 (67)
50–69	n (%)	382 (92)	32 (8)
70-	n (%)	233 (97)	8 (3)

### Assistive devices and gait speed

Results of the use of walking aids are presented in Table 4. Five percent (n = 41) of the patients used a ventilator, 38 patients from the Nordic region and 3 patients from outside the Nordic region.

**Table 4:** Patients use of walking aids divided into “Nordic region” (n = 622) and “Outside the Nordic region” (n = 180).

Walking aids		Nordic region	Outside the Nordic region
No walking aids	n (%)	399 (84)	79 (17)
Crutches/cane or walker	n (%)	151 (69)	68 (31)
Wheelchair sometimes	n (%)	73 (80)	18 (20)
Wheelchair always	n (%)	39 (72)	15 (28)

At 30 m Walk Test, the patients walked in average 71% slower than a sample of the normal population (mean 0.96 meter/second) in self-selected speed (n = 680) and 61% slower than normal population (mean 1.2 m/s) in maximal speed (n = 667). When comparing the patients maximal walking speed in percent of the normal population, women with polio walked 64% as fast as the normal population compared with men who walked 58% as fast as the normal population, the difference reached statistically significance (*p* = 0.001). There were no differences between genders regarding self-selected walking speed (*p* = 0.358). When comparing the patients walking speed in percent of the normal population, patients from the Nordic region walked faster than patients from outside The Nordic region in both self-selected (74% and 61% respectively) (*p*<0.001) and maximal walking speed (64% and 49% respectively) (*p*<0.001).

### Muscle strength of the knee

Results of knee muscle strength from all patients measured, regardless leg/s affected by polio or not, compared to normal population see Table 5. In isometric- and isokinetic knee muscle strength, patients with polio are about 60–70% as strong as a sample of the normal population. Patients have generally more endurance compared to the normal population in isometric knee muscle endurance.

**Table 5:** Knee muscle strength in percent of the normal population.

		Right		Left	
		n	mean (SD)	n	mean (SD)
Isometric (Nm)	Ext	552	61 (34)	547	65 (36)
	Flex	585	71 (40)	567	71 (39)
Isokinetic (Nm)	Ext	504	68 (32)	503	70 (34)
	Flex	548	70 (37)	529	71 (36)
Isometric endurance (s)		442	112 (67)	444	114 (67)

Nineteen percent and 19% (right and left respectively) of the patients with no result in isometric knee extension strength tried to perform the test but were too weak to get a result, 8 and 12% (right and left respectively) tried to perform the test but were too weak to get a result of isometric knee flexion strength. Twenty-two percent and 22% (right and left respectively) of the patients with no result in isokinetic knee extension strength tried to perform the test but were too weak to get a result, 14 and 19% (right and left respectively) tried to perform the test but were too weak to get a result of isokinetic knee flexion strength. One percent (right and left) of the patients with no result in isometric endurance tried to perform the test but were too weak to get a result.

**Fig 2:**
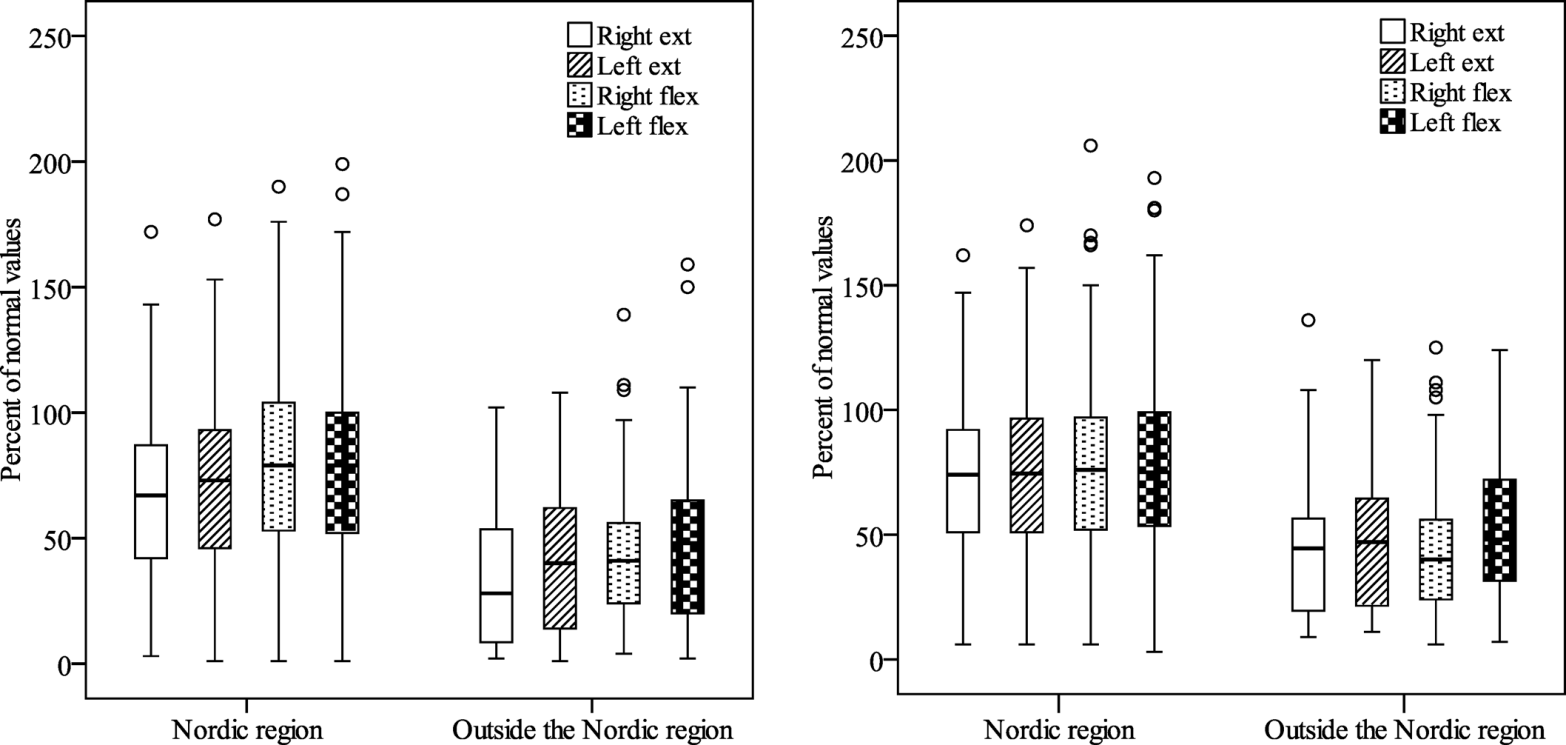
Results of isometric (left “fig, A”) and isokinetic (right “fig, B”) knee extension and flexion strength at 60° knee angle, right and left leg, for patients with polio in both legs. Results are presented in percent of normal population for patients from “Nordic region” (isometric/isokinetic n = 306/268) and “Outside Nordic region” (isometric/isokinetic n = 55/32). The bottom and top of the box are the first and third quartiles, and the band inside the box is the median (second quartile). The ends of the whiskers represent the lowest data and the highest data that is not an outlier. Any data not included between the whiskers are plotted as an outlier and are more than 1.5 box-lengths from the first and third quartiles respectively.

Results of isometric and isokinetic knee muscle strength in percent of the normal population for patients with both legs affected by polio (according to clinical classification of polio) are presented in Fig 2. When comparing the patient’s knee muscle strength in percent of the normal population, no differences were seen between genders. When comparing the patients knee muscle strength in percent of the normal population, patients from the Nordic region were stronger than patients from outside The Nordic region in both isometric (63–75% and 35–45% respectively) (*p*<0.001) and isokinetic (69–74% and 44–51% respectively) (*p*<0.001) knee muscle strength as well as isometric endurance (113–120% and 95–106% respectively) (left leg *p* = 0.001, right leg *p* = 0.016).

An increase in maximal isometric knee muscle strength with the length of recovery is seen when data from all patients measured, regardless leg/s affected by polio or not, is presented (see Table 6).

**Table 6:** The recovery of maximal isometric knee strength, measured as peak % of maximal result. Measured every minute (after 1, 2, 3, 4 and 5 minutes). Right n = 429, left n = 428.

	Right					Left				
	1 min	2 min	3 min	4 min	5 min	1 min	2 min	3 min	4 min	5 min
Mean (SD)	84 (12)	88 (9)	91 (9)	92 (9)	93 (9)	83 (12)	88 (10)	91 (9)	92 (9)	94 (9)
Min/Max	34/100	44/100	41/100	51/104	49/113	29/102	39/105	41/102	33/102	40/104

### Muscle strength of the foot

Results of muscle strength of the foot from all patients measured, regardless leg/s affected by polio or not, compared to normal population see Table 7. In dorsal- and plantar flexion strength, patients with polio are about 50–65% as strong as a sample of the normal population.

**Table 7:** Results of dorsal- and plantarflexion ankle strength (Nm) in percent of the normal population.

	Right		Left	
	n	mean (SD)	n	mean (SD)
Dorsalflexion	409	65 (38)	409	62 (34)
Plantarflexion	500	53 (31)	486	59 (32)

Twenty-seven to thirty percent (right and left respectively) of the patients with no result in dorsal flexion strength tried to perform the test but were too weak to get a result, 7 and 10% (right and left respectively) tried to perform the test but were too weak to get a result of plantar flexion strength.

When analyzing muscle strength of the foot in percent of the normal population for patients with both legs affected by polio (according to clinical classification of polio), women were stronger than men in dorsal flexion strength (65–66% of normal values and 50–56% respectively) reaching statistically significance (left leg *p*<0.001, right leg *p* = 0.034). When comparing the patient’s muscle strength of the foot in percent of the normal population, patients from the Nordic region were stronger than patients from outside The Nordic region in both plantar (54–61% and 41–44% respectively) (*p*<0.001) and dorsal flexion strength (64% and 42–47% respectively) (*p*<0.001).

### Grip strength

Results of muscle strength of hand from all patients measured, regardless arm/s affected by polio or not, compared to normal population see Table 8. In maximal hand grip strength and isometric hand grip strength during 10 seconds, patients with polio are about 70–80% as strong as a sample of the normal population.

**Table 8:** Grip strength (N) presented in percent of the normal population.

	Right		Left	
	n	mean (SD)	n	mean (SD)
Maximal	520	74 (25)	518	77 (25)
Isometric, during 10s	520	69 (25)	518	72 (26)

Almost everyone who tried to perform the grip strength test of the hand could perform the test and get a result.

When analyzing the patient’s grip strength in percent of the normal population for patients with both arms affected by polio (according to clinical classification of polio), no differences were seen between genders. When comparing the patient’s grip strength in percent of the normal population, patients from the Nordic region were stronger than patients from outside the Nordic region in both isometric grip strength (68–73% and 55–60% respectively) (left *p* = 0.007, right *p* = 0.006) and maximal grip strength (77% and 65% respectively) (left *p* = 0.011).

## Discussion

This study describes characteristics as well as gait and muscle strength of 842 polio patients visiting a polio clinic in Sweden during almost 20 years. The high number of patients included and the large amount of data makes this into a unique material and the largest study of its kind. As all patients from the clinic were included, we have no reason not to believe that the patients are representative for an average polio patient group and thereby comparable with polio patients visiting other polio clinics in Sweden and probably outside Sweden as well. The amount of immigrants, and where they emigrated from, may however differ from country to country.

More than twenty percent of the patients were from countries outside the Nordic region. Most of the patients from the Nordic region were older than 50 years i.e. they are in working age and will be so for many years to come. The opposite was seen for patients outside the Nordic region. This was, however, expected as Europe has been polio free since more than 10 years. As polio is still not eradicated, even endemic in some countries and patients with polio immigrating to Sweden are in working age, the need for multi professional teams working with polio rehabilitation in Sweden are of great importance, and will be so for many years to come. The younger patients with polio emigrating from countries with different cultures may also lead to a challenge for the multi professional teams and are of importance when planning for the care of polio patients the coming years.

Women were dominating the group of patients from the Nordic region. From outside Europe, on the other hand, there were more men than women affected by polio included in the study. One may speculate that more men than women with polio manage to immigrate to Sweden and/or more men than women consult Swedish healthcare and thereby polio clinics. This, however, need some further research.

Data of body parts affected by polio were equal both between genders and between origins (as shown in Table 2). There were, however, discrepancies in walking speed and muscle strength in patients from different origins. Patients from the Nordic region walked faster and were stronger in several of the muscle tests, than patients from outside the Nordic region, when data were compared with normalized for age and sex. One should note that the control values are based on a population living in Sweden but no information on origin of the persons is available. Furthermore, patients from outside the Nordic region used crutches, cane or walker to a greater extent compared to patients from the Nordic region. The use of wheelchair were, however, equal between the two groups of patients. One may speculate if the virus affected patients in other parts of the world may be a virus causing paralytic polio to a greater extent. Another, maybe more likely, explanation may be that the Nordic patients have received more and better care in the acute phase of polio as well as more information, aids and exercise both in the acute phase and in the aftermaths compared to the patients from countries outside the Nordic region. There might also be different attitudes towards exercise in different cultures. A greater percentage of the patients from the Nordic region were using a ventilator compared to patients from outside the Nordic region. An explanation can be that the patients most affected by polio, involving the respiratory function, might not survive in countries with poorer opportunities for advanced acute care of these patients.

Polio in lower extremities was more common than polio in upper extremities, verified both by EMG and clinical classification. This is in accordance with earlier studies. Polio in lower extremities was also classified as clinically unstable or severely atrophic to a higher extent than polio in upper extremities (as shown in Fig 1). This is in accordance with an earlier study by Sandberg et al [20] indicating a more pronounced ongoing denervation-reinnervation process over time in a lower extremity muscle compared to upper extremity muscle (tibialis anterior and biceps brachii respectively). The same pattern was also seen in patients studied in Minnesota where patients with leg weakness were twice as likely to complain of new problems compared to those with arm weakness [10].

The ongoing denervation-reinnervation process in patients with PPS results in larger motor units. When motor-unit size has reached an upper limit, further losses of neurons can no longer be compensated for and this results in increased muscle weakness [21]. The patients showed to be stronger in isometric endurance compared to normal population. This may be explained by the fact that the patients were weaker than the normal population in isometric peak torque, which the measure of isometric endurance was based on. An increase in type I (slow) muscle fibres has also been described in prior polio patients [22–23] and may be due to a transition of type II (fast) to type I (slow). The patients did not recover completely within five minutes. However they recovered from eighty to ninety percent from the first to the last test. It would be of interest to see after how long time patients with polio were completely recovered compared to normal population.

An important study limitation were seen in the classification of polio as polio were classified for left and right arm and leg, respectively, and not per muscle group. This can explain why a polio affected leg in some cases was stronger compared to normal values as the muscles involved in the strength measured i.e. knee flexion and knee extension muscles may not be affected of polio. And the same is applicable regarding muscle strength of the foot as well as grip strength. Data of muscle strength from some of the subjects were missing for different reasons i.e. they may have just not had time to participate, or refused to participate and some were too weak to perform the strength test. Some of the patients with muscle strength data missing, tried to perform the test, but were too weak to get a result. In the future, the use of ultrasound may be used to assess muscle function [24]. This would give the possibility to have more information of muscle function since this does not require that the patient has muscle strength to overcome gravity, which is a requirement for isokinetic testing.
